# Exploring Mexican psychotherapist's attitudes towards knowledge and use of serious games in clinical practice

**DOI:** 10.3389/fdgth.2025.1584171

**Published:** 2025-07-11

**Authors:** Marcela Tiburcio, Nora Angélica Martínez-Vélez, Rosalía Pilar Bernal-Pérez, Jessica Huss, Christiane Eichenberg

**Affiliations:** ^1^ Department of Social Sciences in Health, Direction of Epidemiological and Psychosocial Research, Ramón de la Fuente Muñiz National Institute of Psychiatry, Mexico City, México; ^2^ Department of Psychology, University of Kassel, Kassel, Germany; ^3^ Institute for Psychosomatics, Faculty of Medicine, Sigmund Freud Private University, Vienna, Austria

**Keywords:** serious games, psychotherapy, attitudes, e-mental health, Latin America

## Abstract

**Objective:**

To explore the perceptions, attitudes, and use of serious games (SG) in psychotherapeutic intervention from the perspective of psychotherapists in Mexico.

**Methods:**

An online survey was conducted using snowball sampling through social media. The participation of psychotherapists was sought, regardless of their theoretical approach. The questionnaire, available through SurveyMonkey, explored demographics, experience with electronic devices and computer games, experience with SG, and attitudes toward SG use. Two hundred and sixteen health professionals participated, yielding 135 (62.5%) complete questionnaires; 74.1% of the respondents were women. Participants had an average age of 35 (SD + 11) and 9.5 (SD + 8.5) years of clinical experience. The most common psychotherapeutic approach was cognitive-behavioral (66.7%).

**Results:**

Nearly all respondents used a technological modality as part of psychotherapy but only nine (6.6%) reported using SG. Participants considered that SG could be used to treat anxiety and emotional and impulse control disorders with a mild to moderate degree of severity. A total of 4.8% of therapists showed unfavorable attitudes and 9.8% highly favorable attitudes towards SG; no statistically significant differences were observed by sex, age, years of experience, or psychotherapeutic approach. Although SG are a little-known care modality in Mexico, some potential benefits have been acknowledged, particularly in the care of adolescents and young people, for specific skills training. More information on their advantages and disadvantages should be made available to those seeking care and health professionals.

## Introduction

Digital games, with their engaging and motivating attributes, have gained popularity beyond entertainment and are increasingly used in educational and health-related contexts. Within this broader category, Serious Games (SG) are purposefully designed to promote learning, develop cognitive skills or support behavioral change (1). A growing body of international research supports their effectiveness in various fields, including education (2–4) and mental health (5–9). In the context of psychotherapy, SG have been studied in high-income, English-speaking countries (10) where evidence highlight their usefulness for skill training, symptom management, and complementing therapeutic processes (5, 9).

In Latin America, SG research is still emerging and largely focused on educational or cognitive training rather than on clinical applications (11). For instance, Rybarczyk (12) examined a SG to strengthen phonological awareness in Ecuadorian children with neurodevelopmental disorders. In Peru, Ocaña and Andrade (7) adapted the SCRUM framework (SCRUM-SUM) to guide SG development for mental health purposes, enhancing accessibility and user engagement.

However, such interventions remain at an early stage and are rarely integrated into clinical practice. In Mexico, a few SG initiatives have addressed health and developmental needs in children. A study showed high agreement between user satisfaction and the interface tested for a SG developed to support problem-solving in children with ADHD (2). Mercado and collaborators (13) created *FarmerKeeper*, a neurofeedback-based SG for children with autism, which improved attentional engagement during sessions. Another example is *FoodRateMaster,* a game to promote healthy eating behaviors among children, showing potential to influence dietary choices (4). While promising, these games are not embedded in psychotherapeutic contexts nor widely used by clinicians.

A review of 24 studies in Spanish-speaking low and middle-income countries (LMICs) in Latin America found that SG and video games have been used to address emotional disorders, anxiety, depression, anger management, impulse control problems, cravings, and post-traumatic stress disorder symptoms (PTSD) among others. Although most studies reported positive outcomes, they rarely included rigorous clinical evaluation (14).

The novelty of SG in therapy has also brought challenges. Some studies report negative attitudes or stigma toward their use, while on the others highlight their potential as valuable tools in health care (15). Encouragingly, therapists' perspectives appear to be evolving. Miloff et al. (16) observed a growing openness to integrating technologies like virtual reality into routine clinical practice. In Germany, Eichenberg et al. (6) emphasized the potential of SG in psychotherapy but also the need to better understand patients' and therapists' familiarity and willingness to use them. Indeed, therapists play an important role in facilitating the adoption of e-mental health tools among their patients (17–19).

Despite the evidence of SG in mental health care and its imminent development and growth, adoption in Latin American health care remains limited. Reported barriers include regulatory uncertainty, technological and financial constraints, and concerns about impacts on the therapeutic alliance (16, 20). Additionally, research has largely focused on users or developers, leaving therapists' perspectives underexplored.

In Mexico, little is known about how psychotherapists perceive SG, whether they use them, and what barriers or facilitators they encounter. Addressing this knowledge gap is essential to inform the development and integration of SG into local mental health services.

This study aims to explore Mexican psychotherapists' perceptions, knowledge, experience, and attitudes toward the use of SG as part of the clinical practice.

## Method

This study is part of a larger international project exploring the acceptability of SG in different countries (10). The broader study included countries with presumably low awareness of electronic mental health (e-MH) practices as well as pioneering ones that have already implemented e-MH into national health strategies (21), encompassing ten high-, middle-, and low-income countries (10). The present report focuses exclusively on data collected from Mexico.

### Study design

This was an exploratory, descriptive study undertaken through an online survey. A snowball sampling method was used to explore the attitudes and perceptions of psychotherapists living in Mexico towards using SG as part of the therapeutic process. The survey explored the extent of participants' knowledge and practical experience with SG, their opinion about their clinical utility, the requirements they consider necessary to use SG in clinical practice, and the types of patients and clinical conditions appropriate for such interventions.

### Participants

The study addressed psychotherapists living in Mexico with no restrictions on their theoretical approach. Psychotherapists in training were also eligible. Recruitment was conducted via professional psychology associations, psychology schools, and social media platforms such as Facebook, LinkedIn, and X, formerly known as Twitter. Exclusion criteria included: (a) not being in the country of interest, (b) not being currently involved in clinical practice, and (c) providing incomplete or incongruent answers.

### Instrument

The survey instrument was originally developed by Eichenberg et al. (6) to assess knowledge, use, and intent to use SG within psychotherapy. The questionnaire contained 46 closed and open-ended questions on demographic data, professional experience, theoretical approach, experience with electronic devices and computer games, experience and knowledge of SG. The survey also included a scale of attitudes towards the use of SG, consisting of sixteen Likert- type items with five response options ranging from “totally disagree” to “totally agree”. The reliability coefficient for the overall scale in this sample was 0.839. The questionnaire took approximately fifteen minutes to complete.

### Procedure

The questionnaire was administered using the SurveyMonkey platform from November 1, 2019, to June 30, 2020. The invitation and survey link were published on social media (Facebook, Twitter, and WhatsApp). The research protocol was approved by the Ethics Committee of the Sigmund Freud University and the Research Ethics Committee of the Ramón de la Fuente Muñiz National Institute of Psychiatry (CEI/C/043/2019). All participants provided informed consent prior to participation. To ensure the confidentiality and anonymity, no personal data was requested; instead, identification codes were created.

### Data analysis

Descriptive statistics (frequencies and percentages by sex) were calculated for sociodemographic and other numerical variables Qualitative data from the open-ended survey questions were analyzed using category-based content analysis, following the method proposed by Miles and Huberman (22). The process involved three interrelated phases: data reduction, descriptive analysis and interpretation. Responses related to attitudes and use of SG were classified into two main categories: barriers and facilitators, each further divided into thematic sub-categories of analysis.

## Results

### Sample description

Of the 216 health professionals who began the survey, 135 (62.5%) met the inclusion criteria and completed the full questionnaire. Most respondents were women (74.1%), with a mean age of 35 years (SD ± 11) and an average of 9.5 years of clinical experience (SD ± 8.5). Nearly half reported using a cognitive behavioral approach and primarily working with adults in private practice. Over 60% conducted individual therapy and were actively engaged in psychotherapeutic work (Table 1).

**Table 1:** Clinical experience and work context by sex.

Variables	Men		Women		Total		
	(*n* = 35)		(*n* = 100)		(*n* = 135)		
	*f*	*%*	*f*	*%*	*f*	*%*	X^2^/ *df*
Theoretical approach							
CBT	19	55.9	47	50.5	66	52.0	0.285/1
Other^a^	15	44.1	46	49.5	61	48.0	
Therapy experience							
1–5 years	16	51.6	42	45.2	58	46.8	3.661/2
6–10 years	8	25.8	14	15.1	22	17.7	
11 or more years	7	22.6	37	39.8	44	35.5	
Area of specialization							
Children and adolescents	5	16.7	24	26.7	29	24.2	1.449/2
Adults	15	50.0	43	47.8	58	48.3	
Both	10	33.3	23	25.6	33	27.5	
Type of therapy							
Group	-	-	7	7.0	7	5.2	3.089/2
Individual	23	65.7	67	67.0	90	66.7	
Both	12	34.3	26	26.0	38	28.1	
Area of Work							
Prevention	-	-	3	3.0	3	2.3	1.591/3
Consultation	5	14.7	15	15.2	20	15.0	
Psychotherapy	25	73.5	65	65.7	90	67.7	
Rehabilitation	4	11.8	16	16.2	20	15.0	
Which age groups do you work with?							
Children	-	-	6	6.0	6	4.4	6.017/3
Adolescents	6	17.1	6	6.0	12	8.9	
Adults	23	65.7	66	66.0	89	65.9	
Older adults	6	17.1	22	22.0	28	20.7	
Where do you work?							
Clinic/Hospital	4	11.8	16	16.8	20	15.5	4.354/4
Private practice	17	50.0	55	57.9	72	55.8	
Consultancy	5	14.7	7	7.4	12	9.3	
Research	8	23.5	14	14.7	22	17.1	
Currently not working	-	-	3	3.2	3	2.3	

^a^ CBT, humanistic, psychoanalytic, systemic.

### Knowledge and perceived uses of SG

A large majority of participants (94.8%) reported having played video games at least once in their lives, 56.3% had done so on a personal device. In terms of their knowledge of SG, 63.7% rated it at a beginner's level. Only nine professionals (6.6%) reported having used SG in clinical practice. Among these, SPARX^1^ was the most frequently mentioned, followed by The Journal, E-couch and Beating the Rules.

**Figure 1:**
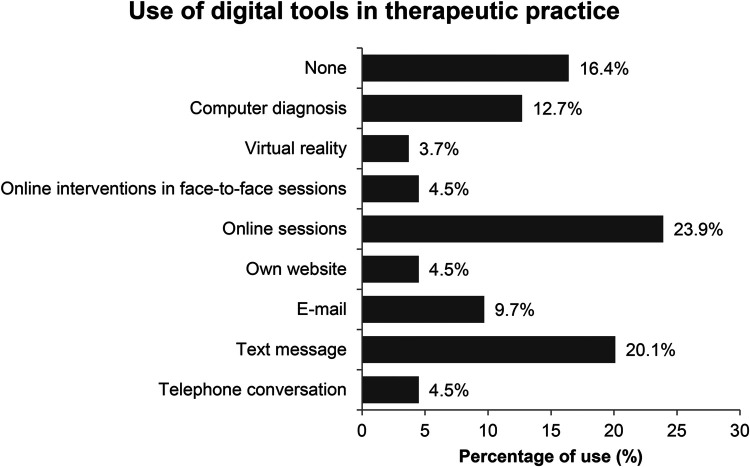
Use of digital tools in therapeutic practice.

Participants were asked about the digital methods they use in their clinical work. The most frequently reported tools were online sessions (23.9%) and text messaging (20.1%), followed by computer-assisted diagnosis (12.7%) and email communication (9.7%). Less common were online interventions during face-to-face sessions (4.5%), the use of a personal website (4.5%), and telephone conversations (4.5%). Only 3.7% of participants reported using virtual reality, and 16.4% indicated that they do not use any digital tools in their therapeutic practice. These results suggest that while digital tools are being integrated into psychotherapy, their use is still limited among a notable portion of professionals (Figure 1).

Participants consider that SG can be used at the request of users (35%) and after patients have been discharged (36.7%). Over a third of professionals consider that SG should be open access or provided by the clinician (32.6% and 42.2%, respectively); 37% consider SG would be most suitable for treating adults (37%) or young adults (26.7%). Regarding the type of psychological problems, 40% of participants considered SG appropriate for treating personality disorders, 23.9% for adjustment disorders, and 20.1% for impulse control disorders. Over half the participants consider that SG should preferably be used with patients with moderate problems.

Participants indicated that SG progress should be monitored primarily through feedback, either provided directly to patients (36.6%) or through other people (49.3%) (Table 2).

**Table 2:** Knowledge and perceived uses of SG.

Variables	Men		Women		Total		
	(*n* = 35)		(*n* = 100)		(*n* = 135)		
	*f*	*%*	*f*	*%*	*f*	*%*	X^2^/ *df*
How do you imagine Serious Games being used?							
For prevention	-	-	1	1.1	1	0.8	3.095/4
As an adjunct to psychotherapy	5	16.7	26	28.9	31	25.8	
To provide support for users after discharge	11	36.7	33	36.7	44	36.7	
Instead of psychotherapy	1	3.3	1	1.1	2	1.7	
At the request of the user	13	43.3	29	32.2	42	35.0	
How should users access SG?							
Online	1	2.9	4	4.0	5	3.7	1.505/3
Without the need for internet	10	28.6	19	19.0	29	21.5	
Through the clinician	13	37.1	44	44.0	57	42.2	
Open access	11	31.4	33	33.0	44	32.6	
How should SG progress be monitored?							
Direct access to SG	5	14.7	10	10.0	15	11.2	2.336/3
Patient feedback	15	44.1	34	34.0	49	36.6	
Feedback from others	13	38.2	53	53.0	66	49.3	
Progress should not be monitored	1	2.9	3	3.0	4	3.0	
What psychological problems can SG be used for?							
Somatic diseases	-	-	2	2.0	2	1.5	10.456/9
Emotional disorders	1	2.9	-	-	2	1.5	
Anxiety	-	-	1	1.0	1	0.7	
OCD	-	-	3	3.0	3	2.2	
Eating disorders	3	8.6	4	4.0	7	5.2	
Sleep-wake disorders	-	-	3	3.0	3	2.2	
Post-traumatic stress	-	-	4	4.0	4	3.0	
Impulse control disorders	5	18.5	22	22.2	27	20.1	
Adjustment disorders	11	31.4	21	21.2	32	23.9	
Personality disorders	15	11.2	39	39.4	54	40.3	
Severity of the disorder you would treat with SG?							
Mild	4	11.4	21	21.0	25	18.5	2.995/2
Moderate	26	74.3	58	58.0	84	62.0	
Severe	5	14.3	21	21.0	26	19.3	
What age group would you use SG with?							
Children	1	2.9	1	1.0	2	1.5	1.279/4
Teenagers	3	8.6	9	9.0	12	8.9	
Young adults	11	31.4	25	25.0	36	26.7	
Adults	12	34.3	38	38.0	50	37.0	
Older adults	8	5.9	27	27.0	35	25.9	

### Attitudes towards SG

Therapists' attitudes towards SG were explored by analyzing their perceptions of facilitators and barriers to implement SG in clinical settings.

Table 3 presents the facilitators, while Table 4 outlines the barriers associated with the use of SG in psychotherapy.

**Table 3:** Facilitators to the acceptability of SG in psychotherapy.

Category	Testimonials
Interest in learning	“I find this new topic interesting. All mental health professionals should become familiar with it”
	“It is important to disseminate them more widely and train those who do not know/use them”
	“It's an area of opportunity and it can achieve positive results, provided the professional is trained to use them”
Innovation and generational fit	“It is a useful tool for children and adolescents who are digital natives”
	“It is an innovative tool with enormous potential for the future that will enable us to optimize resources”
	“It could boost treatment adherence with adolescents and young people who are often on the internet”
Receptiveness to training and dissemination	“I didn't know these games existed. I think it's an interesting topic, and I hope it is a field that will expand in the coming years”
	“I would like to know how they can be implemented at the institutional level”
	“With proper training [this intervention] could be successfully implemented”
Clinical potential	“We should leverage technological advances to enable the patient to act as their own guide in the therapeutic process”
	“It could enhance students' social and emotional skills”
	“It would be an excellent way to reach a larger population in terms of psychological services, saving time and facilitating access to patients due to the practicality of these tools”

**Table 4:** Barriers to the acceptability of SG in psychotherapy.

Category	Testimonials
Limited access to technology	“Not all populations that go to an institution have the resources to access the internet”
	“It would require the installation of the program that would be used, the purchase of equipment, a camera and a microphone”
	“Institutions take a long time to update equipment. This could make it difficult to manage the information and limit the results of the intervention”
Risk of overuse or addiction	“Continuous use can create another disorder due to excessive cell phone use”
	“It could be more of a distraction than a tool for paying greater attention to their (the patients') problems”
	“It could promote gambling”
Lack of digital literacy	“It could be difficult for patients who are unfamiliar with the devices and create resistance”
	“It can be more complicated for older adults”
	“Many patients not knowing how to use the tools”
Clinical concerns	“Low therapeutic control and supervision, lack of commitment and seriousness. Close contact with the patient is lost. No limits established. The process becomes impersonal”
	“Artificial intelligence is not yet sufficiently developed to detect the subtleties of clinical judgment”
	“Difficulty assessing key behaviors”
Lack of familiarity with SG	“I am not familiar with them”
	“I have no experience with SG”

Data reveal a generally positive outlook toward incorporating SG into therapeutic work. In this regard, a central positive aspect participants mentioned was using technology as an ally, a valuable complement to psychotherapy that can help reduce costs and increase accessibility. SG were seen as a promising tool, particularly for new generations, and potentially helpful for improving treatment adherence among digital natives.

One of the most striking findings was their willingness to learn more about SG, expressing interest in understanding what SG are, how they work and how they can be implemented in specific clinical contexts. Therapists are convinced that it is not only desirable to adopt new ways of working linked to technology, but that technology is required to meet the needs of the current clinical practice.

The acceptability of SG is also shaped by various concerns therapists perceive as obstacles. One of the most frequently mention barrier is the lack of information on SG and their inaccessibility, either for technological reasons or because people do not know how to interact with them. Another aspect that emerged was the lack of clinical sensitivity provided by face-to-face contact with the patient during the therapeutic process; therapists were apprehensive about losing control of the therapeutic process.

Psychotherapists, particularly those working in government settings, expressed concern about the delay in administrative management times, both in regard to the purchase of infrastructure and the authorization required for the adoption of these new technologies.

## Discussion

This study aimed to explore the knowledge and acceptance of therapists of SG use in the mental health field in Mexico. The results obtained show that there is a general willingness and openness on the part of therapists to know what SG are and how to use them in the context of psychotherapy. Key elements in the qualitative information were the willingness to learn about this therapeutic tool, its use with the generations of young people and adolescents, and its clinical potential. The results suggest that SG have the potential for future development in mental health interventions.

There is a broad area of opportunity for the acceptance and use of SG. The availability of information and adequate resources are necessary and desirable factors for therapists to create broader therapeutic and reference frameworks and, thereby, consider SG a useful therapeutic tool.

However, due to ignorance and the current lack of information on SG, a significant proportion of therapists are reluctant to use them in the mental health field, citing a series of clinical and operational reasons that would complicate their use in psychotherapy. Other studies found similar results, including the fact that patients lacked adequate supervision in their interaction with SG, and the impossibility of establishing the same bond of trust as in face-to-face interaction with the therapist (15, 16, 20).

These factors concerning the availability of information and adequate SG resources could be resolved depending on the psychotherapist's theoretical approach. Those who adopt a cognitive-behavioral approach are more likely willing to use SG than those using a psychoanalytic or humanistic approach (23). This is due to how the therapeutic approach conceives the relationship established with the patient (6, 24).

One of the cultural characteristics of the Mexican population is the tendency towards collectivity (25), in other words, people find it easy to spend time together and work in groups. Nevertheless, they also show interest in learning to use technological tools which, despite being more focused on the individual, can pay off by serving a larger population, since they are practical, innovative and, like other online games, SG in particular could also be set up as group games (parent, together with the therapist, together with other affected people—as an idea and suggestion for further game developments).

Regarding infrastructure and availability of resources, unlike high-income countries, such as Canada, which has an Action Plan on Mental Health (26), or China, whose government promotes technology use in health care access (27), Mexico has a different perspective on public health resources. The budget assigned to mental health accounts for just 1.3% of the health budget (28) while the adoption of digital alternatives has been slow.

Knowledge and use of SG in Mexico still need to be improved. In this sample, only 25% of psychotherapists mentioned knowing them, and just nine out of 135 had used them in clinical practice. These results are slightly lower than those reported in the broader international study involving thirteen countries, including Mexico, where 26% of mental healthcare professionals (*N* = 1,497, 70% female) were familiar with the general concept of SG and only 6% had used SG with their patients or clients. The majority (approximately 80%) demonstrated high usage intentions of SG within psychotherapy if given the opportunity (10). Therapists tend to view SG primarily as adjuncts to psychotherapy rather than standalone therapeutic tools (29). They also consider them more appropriate in mild and moderate clinical cases but not in cases of severe psychopathology (19.3%). This finding contradicts the reports of other studies that have developed SG for psychiatric pathologies (30, 31).

The results suggest that the acceptability of SG is constrained by various factors, such as accessibility, information about what they are and how to use them, technological resources, such as computer equipment availability, stable Internet access, and SG software acquisition. In other words, SG are accepted, although their actual use in a country like Mexico is still in its infancy.

The data presented in this study were collected before the outbreak of COVID-19 pandemic. However, the pandemic represented a turning point in digital engagement for many health professionals. The pandemic forced many psychotherapists to seek technological resources that would enable them to continue the psychotherapeutic process (32, 33). This abrupt shift to remote modalities, including telepsychology, may have increased therapist's familiarity with and openness to digital tools in general, potentially reducing resistance to more innovative interventions (34, 35). This raises the question of whether this need for remote work modified therapists' knowledge of technological tools may have, such as SG, to provide accompaniment, thereby reducing the gap between the acceptance and use of SG within the context of psychotherapy in Mexico. Future studies could investigate whether the increased digital exposure port-COVID has translated into greater adoption or sustained interest in SG among practitioners.

The results show that there is insufficient knowledge of SG in Mexico, while at the same time, people express willingness and interest in finding out what they involve. This could be an advantage for designing a strategy to adequately inform mental health professionals about SG, their scope, and potential.

It is worth acknowledging certain aspects of the present study that limit the generalizability of results. For instance, the sample was obtained through a non-random snowball sampling method via social media. While this approach was useful in reaching broader and diverse group of psychotherapists, it may have introduced selection bias. Participants who were already interested in digital tools or more active online may have been more likely to respond. Additionally, the sample shows a bias toward the cognitive-behavioral approach, limiting the points of view of professionals from other therapeutic orientations.

In addition, some studies suggest that online surveys may have slightly lower validity but are generally equivalent to traditional paper-and-pencil surveys (36, 37). Other analyses highlight the various advantages of online surveys, including lower costs, reduced time for administration, easier recruitment, participant anonymity, access to large population samples, and quick data storage.

However, online surveys also face specific challenges, such as participant dropout, which can affect the representativeness and generalizability of the results (38). It has been found that approximately 10% of participants abandon online surveys after the first 12 questions, with a dropout rate ranging from 2%–13% for every additional 100 questions. Notably, abandonment does not correlate with age or gender (39). Instead, factors such as interest in the research topic and the participants' willingness to contribute may influence whether they complete the questionnaire (40, 41).

To reduce dropout rates in future online surveys, it is advisable to implement strategies such as sending prior notifications, issuing email invitations, designing surveys to be simple and concise, and providing incentives for completion (36, 38, 42).

Although there are a few studies on the pedagogical and educational applications of serious games (SG) in Mexico, this study is the first to examine their acceptability in a clinical context. It sets a precedent for further exploration of their applicability and effectiveness in the mental health field and underscores the need for expanded research in this area.

## Data Availability

The raw data supporting the conclusions of this article will be made available by the authors, without undue reservation.

## Footnotes

^1^SPARX is a digital platform that delivers an online intervention designed to help young people manage depression and anxiety. It combines cognitive-behavioral therapy techniques with role-playing game elements to make therapy more accessible and engaging.
